# Screening and validation of lymph node metastasis risk-factor genes in papillary thyroid carcinoma

**DOI:** 10.3389/fendo.2022.991906

**Published:** 2022-11-07

**Authors:** Qiaoyue Zhang, Jing Li, Hengyan Shen, Xinyu Bai, Tao Zhang, Ping Liu

**Affiliations:** ^1^ Department of Clinical Pharmacy, Key Laboratory of Basic Pharmacology of Guizhou Province and School of Pharmacy, Zunyi Medical University, Zunyi, China; ^2^ Liaoning Academy of Traditional Chinese Medicine, Liaoning University of Traditional Chinese Medicine, Shenyang, China; ^3^ Department of Laboratory Medicine, Affiliated Hospital of Zunyi Medical University, Zunyi, China

**Keywords:** papillary thyroid carcinoma, iodine, bioinformatics, clinical samples, transfer of risk genes

## Abstract

**Background:**

Although most papillary thyroid carcinoma (PTC) cases have a good prognosis, some PTCs are more aggressive and are often accompanied by lymph node (LN) metastasis, a high recurrence rate, and poor prognosis. Distinguishing highly invasive metastatic PTC is an urgent problem that needs to be addressed clinically. We analyzed a microarray of metastasized PTC and validated it using quantitative reverse transcription PCR (RT-qPCR) and immunohistochemistry to identify biomarkers that can be used to assess the risk of PTC metastasis.

**Methods:**

The microarray of metastasized PTC was screened using the Gene Expression Omnibus (GEO) database. The differences between cancer and normal tissues were analyzed using the official GEO tool: GEO2R. Gene expression profile data (GEPIA) were used to verify the expression of differential genes in large samples and to analyze their correlation. The Kaplan–Meier plotter (KM-plotter) database was used for the analysis of genes potentially related to survival. RT-qPCR was used to check the expression of risk factor genes in pathological sections from PTC patients with clinical LN metastasis. Immunohistochemistry was used to verify the expression of core risk-associated genes.

**Results:**

Fourteen PTC metastasis-associated genes were identified. In metastasized PTC, *CLDN1*, *LRP4*, *LRRK2*, and *TENM1* were highly expressed, whereas *DIO1*, *DPP6*, *HGD*, *IPCEF1*, *MT1F*, *SLC26A4*, *SLC26A7*, *SPX*, *TFF3*, and *TPO* were expressed at low levels, compared to expression in normal tissues. *DIO1*, *HGD*, *SLC26A4*, and *TPO* were found to be the core risk genes in the PTC metastatic risk set. Results based on clinical samples showed that the expression differences for metastasis risk-associated genes were consistent with the bioinformatics analysis results.

**Conclusions:**

Fourteen differentially expressed genes (*CLDN1*, *LRP4*, *LRRK2*, *TENM1*, *DIO1*, *DPP6*, *HGD*, *IPCEF1*, *MT1F*, *SLC26A4*, *SLC26A7*, *SPX*, *TFF3*, *TPO*) are associated with an increased risk of PTC metastasis, and *DIO1*, *HGD*, *SLC26A4*, and *TPO* are the key risk-associated genes in this set that might affect the occurrence and development of PTC through iodine metabolism. These genes could provide a reference for clinical metastatic PTC risk evaluation and treatment.

## Introduction

The current incidence of thyroid cancer (TC) accounts for 3.4% of all cancers, making it the tenth most prevalent cancer globally and seventh most prevalent in China (1, 2). The reasons for the increased incidence of TC are complex. Overdiagnosis might be one of these, but an actual increase in TC cannot be ruled out (3–5). TC can be distinguished into medullary thyroid carcinoma, anaplastic thyroid carcinoma (ATC), and differentiated thyroid carcinoma (DTC), based on the histological type. DTC can be further distinguished into follicular thyroid cancer (FTC) and papillary thyroid carcinoma (PTC). PTC accounts for approximately 95% of TC cases (6). Patients with early PTC have a 5-year survival rate >90% after conventional treatment, and these are generally considered “indolent tumors” (7, 8). The recurrence rate of PTC is up to 30%, but the survival rate in patients with recurrence and metastasis is significantly reduced (9). Recurrence and metastasis are the key factors involved in PTC-associated mortality. Early detection and active intervention for PTC with a risk of metastasis is an effective strategy for improving prognosis. Unfortunately, a morphological evaluation of the primary tumor cannot be used to predict the likelihood of disease metastasis or recurrence.

Currently, fine-needle aspiration is the most reliable diagnostic tool for PTC; however, it cannot be used to cytologically distinguish between benign and malignant PTC and thus cannot be used to evaluate the risk of PTC metastasis (10, 11). Previous studies have found characteristic genetic changes in PTC, such as *RET*-*PTC*, *NTRK* rearrangements, *RAS* and *BRAF* mutations, and *PAX8*/*PPAR* translocation (12, 13), which have been used for the diagnosis of PTC; however, the genetic changes currently found in PTC cannot be used to characterize tumors with different clinical behaviors, such as LN metastasis (13, 14). Identifying genetic changes that can help diagnose PTC and assess the risk of metastasis is crucial for early clinical intervention in high-risk PTC patients. Therefore, screening for genetic metastatic PTC-specific changes in patients with LN metastasis can provide a reference to assess the risk of PTC metastasis.

The present study aimed to identify prognostic biomarkers for clinical diagnosis and risk assessment with respect to PTC metastasis by screening risk-associated genes exhibiting differential expression. The overall goal of the study was to reduce metastasis and recurrence rates in PTC patients and improve their prognosis.

## Materials and methods

### Microarray data acquisition

The GEO (https://www.ncbi.nlm.nih.gov/geo/) database was searched using the keywords “Thyroid cancer, Human.” Samples comprising metastatic PTC and matching paracancerous tissue were screened from the retrieved results.

### Data processing of DEGs

Using the GEO2R (https://www.ncbi.nlm.nih.gov/geo/info/geo2r.html) online tool, we used a |logFC| > 2 to identify the DEGs between the metastatic PTC and normal thyroid gland tissue samples, and DEGs with P < 0.05 were screened. The raw data were then filtered online using the Venn diagram database to detect DEGs among the three datasets. DEGs with a logFC < −2 and logFC > 2 were considered downregulated and upregulated, respectively.

### Verification of DEGs

GEPIA (http://gepia.cancer-pku.cn/) was used to verify the expression of DEGs with statistically significant differences based on a large sample size.

### Analysis of DEGs associated with survival

The KM-plotter (https://kmplot.com/analysis/) was used to analyze the influence of genes on overall survival (OS) and relapse-free survival (RFS).

### Correlation analysis of DEGs

The obtained DEGs were filtered through the GEPIA database for correlation analysis. The correlation between each DEG in TC was obtained, and the core risk-associated genes in the set of metastasis-associated genes were screened based on these correlations. The R software (version 4.0.3) was used to visualize correlations.

### Validation of risk-associated gene sets

This study was reviewed by the Biomedical Research Ethics Committee of the Affiliated Hospital of Zunyi Medical University, batch no. KLL-2022-448. Preliminary data from major cancer databases represent a large sample size of real patient information. Therefore, in this study, eight clinical samples were collected for validation based on a small clinical sample set. The inclusion criteria were patients with confirmed PTC and metastatic lesions, between 18 and 70 years of age, who visited the Affiliated Hospital of Zunyi Medical University in 2020–2021. Patients with TC subtypes such as PTMC and ATC and other major diseases were excluded from the study. Pathological tissues of metastatic PTC patients meeting the inclusion criteria were collected and pathological sections and paraffin block were produced. The cancerous/paracancerous tissues were isolated from some sections under a microscope for RNA extraction. RT-qPCR was used to verify the 18 genes screened using bioinformatics associated with a risk of metastasis. Immunohistochemistry was performed to confirm the expression of the four core risk-associated genes that were highly associated with each risk gene.

### RT-qPCR

RNA was extracted from tissues from patients with metastatic PTC using an RNeasy FFPE Kit (RNeasy FFPE Kit, QIAGEN, Hilden, Germany). cDNA was then synthesized using a reverse transcriptase system (SureScripTM First-Strand cDNA Synthesis Kit, GeneCopoeia, Guangzhou, China). RT-qPCR was used to validate gene expression in accordance with the manufacturer’s instructions (BlazeTaq SYBR Green qPCR Mix 2.0, GeneCopoeia). The expression levels of all genes were normalized against *ATCB* gene expression, and relative gene expression was calculated using the 2^−ΔΔCT^ method. Primers for RT-qPCR were purchased from Sangon Biotech (Shanghai, China).

### Immunohistochemistry

The tissues from patients with metastasized PTC were fixed with formalin, included in paraffin, and sections were prepared. For the immunohistochemical (IHC) analysis of deiodinase, iodothyronine type I (DIO1, 1:100, Affinity Biosciences, OH, USA), homogentisate 1,2-dioxygenase (HGD, 1:100, Santa Cruz Biotechnology, Dallas, TX, USA), solute carrier family 26 member 4 (SLC26A4, 1:100, Santa Cruz Biotechnology), and thyroid peroxidase (TPO, 1:100, Servicebio, Wuhan, China). Pathological changes were observed under an optical microscope (NI-U, Nikon, Tokyo, Japan), and images were captured with a digital camera (DS-RI2, Nikon).

### Institutional review board approval (or waiver) statement

In accordance with the Biosafety Law of the People’s Republic of China and other laws, regulations, rules, normative documents, and international standards, this study was reviewed by the Biomedical Research Ethics Committee of Zunyi Medical University Hospital; the project was approved and the research conducted according to the scheduled plan (batch number, KLL-2022-448).

### Statistical analysis

The SPSS 22.0 software was used for all statistical analyses. All data are presented as the standard error of the mean and compared using Student’s *t*-test. Statistical significance was set at *P* < 0.05.

## Results

### Microarray data information

The NCBI GEO database included three gene expression profiles that met the requirements (including metastasized PTC and normal paracancerous tissues) as follows: GSE151181 (15) from the GPL23159 platform ([Clariom_S_Human] Affymetrix Clariom S Assay, Human; includes Pico Assay), GSE6004 (16) and GSE60542 (17) from the GPL570 platform [(HG-U133_Plus_2) Affymetrix Human Genome U133 Plus 2.0 Array]. In total, 100 samples met these requirements, consisting of 53 metastatic PTC and 47 normal thyroid tissue samples.

### Identification of DEGs in metastasized PTC

**Figure 1:**
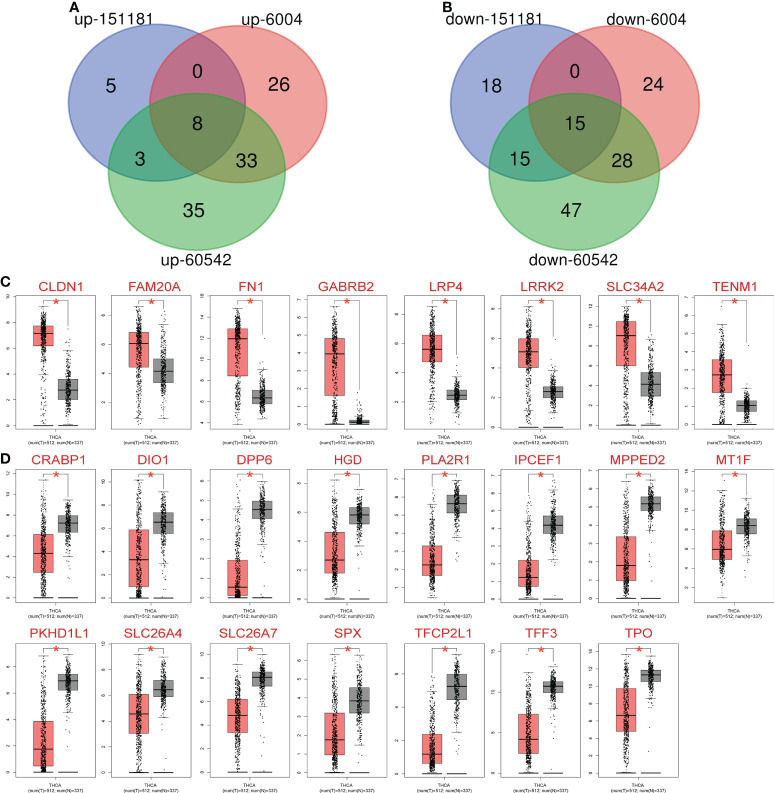
Expression of 23 differentially expressed genes (DEGs) in metastatic PTC and non-cancer tissues. **(A)** Common upregulated intersection DEGs obtained from Gene Expression Omnibus database: GSE15181, GSE6004 and GSE60542. **(B)** Common downregulated intersection DEGs obtained Gene Expression Omnibus database: GSE15181, GSE6004 and GSE60542. **(C)** Upregulated DEGs expression in thyroid cancer (TC) patients and normal controls. **(D)** Downregulated DEGs expression in TC patients and normal controls. Red: Cancer, black: Normal, ^*^P< 0.05 vs. normal group.

The GEO2R online tool identified 462, 1392, and 2026 DEGs from GSE151181 (15), GSE6004 (16), and GSE60542 (17), respectively. A |logFC| > 2 and *P* < 0.05 were used as the condition to filter significant DEGs, and subsequently, Venn diagram online tools were used to determine the intersection of the three datasets to obtain 23 DEGs, including 8 upregulated (**Figure 1A**) and 15 downregulated (**Figure 1B**) genes.

### Verification of DEGs

The 23 identified DEGs were subjected to GEPIA for the verification of differential expression between TC and normal control samples, using a large sample size. Statistically significant differences in expression were obtained for 23 DEGs based on the large sample set (*P* < 0.05, **Table 1**); the 8 upregulated (**Figure 1C**) and 15 downregulated (**Figure 1D**) genes were confirmed.

**Table 1:** Twenty-three differentially expressed genes between normal and thyroid cancer (TC) samples.

Expression	GENE
TC expression was lower than normal (*P*<0.05)	*CRABP1, DIO1, DPP6, HGD, IPCEF1, MPPED2, MT1F, PKHD1L1, PLA2R1, SLC26A4, SLC26A7, SPX, TFCP2L1, TFF3, TPO*
TC expression was more than normal (*P*<0.05)	*CLDN1, FAM20A, FN1, GABRB2, LRP4, LRRK2, SLC34A2, TENM1*

### Analysis of DEGs associated with overall survival

**Figure 2:**
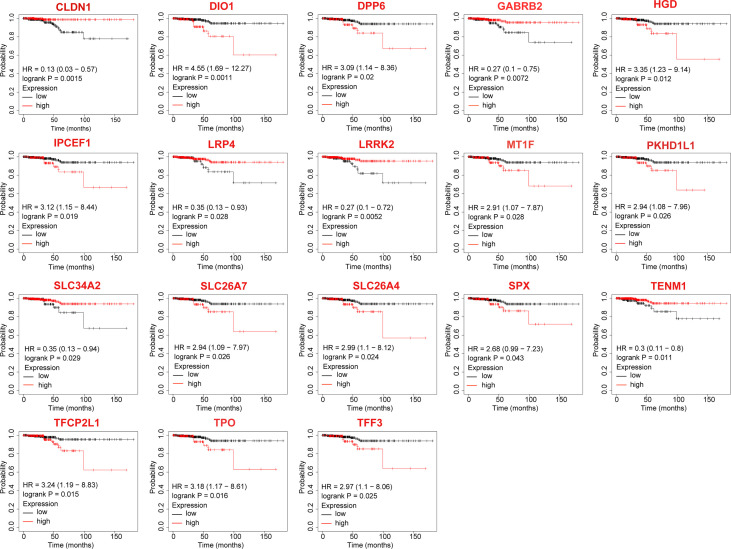
Differentially expressed genes that significantly affect overall survival in thyroid cancer patients.

The KM-plotter was used to analyze the association between OS and the 23 DEGs. Eighteen DEGs were associated with reduced OS in patients with TC (*P* < 0.05, **Table 2** and **Figure 2**), whereas for five the association was not significant (*P* > 0.05, **Table 2**).

**Table 2:** Kaplan–Meier-Plotter analysis of the association between prognosis and 23 differentially expressed genes.

Category	Genes
Significant impact on survival (*P* < 0.05)	*CLDN1*, *GABRB2*, *LRP4*, *LRRK2*, *SLC34A2*, *TENM1*, *DIO1*, *DPP6*, *HGD*, *IPCEF1*, *MT1F*, *PKHD1L1*, *SLC26A4*, *SLC26A7*, *SPX*, *TFCP2L1*, *TFF3*, *TPO*
Without significant impact on survival (*P* > 0.05)	*CRABP1*, *FAM20A*, *FN1*, *MPPED2*, *PLA2R1*

### Correlation analysis of DEGs

**Figure 3:**
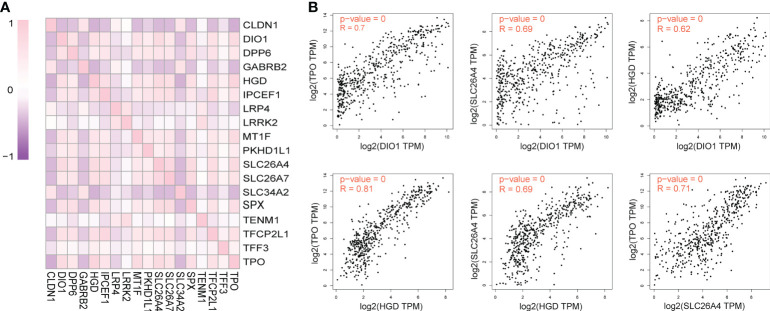
Pairwise correlation of differential expressed genes (DEGs) significantly affecting overall survival of TC patients in the context of TC disease. **(A)** R Software visualization of pairwise correlations of DEGs that significantly affected overall survival. **(B)** The correlation figure of *DIO1* and *TPO*, *DIO1* and *SLC26A4*, *DIO1* and *HGD*, *HGD* and *TPO*, *HGD* and *SLC26A4*, *TPO* and *SLC26A4*.

The 18 DEGs that affected the prognosis of patients might be risk factors for PTC metastasis. Correlation analysis was performed for this risk-associated set using GEPIA, and the results were visualized using the R software (**Figure 3A**). A Pearson correlation coefficient absolute value < 0.4 (very weak correlation) was scored as 0, 0.4–0.6 (weak correlation) was scored as 1 point, 0.6–0.8 (strong correlation) was scored as 2 points, 0.8–1.0 (very strong correlation) was scored as 3 points, and the highest cumulative score was considered the core risk-associated gene. The Pearson correlation coefficient cumulative scores of the 18 DEGs are ranked as follows: *HGD* (21), *TPO* (21), *SLC26A4* (20), *DIO1* (19), *SLC26A7* (18), *SPX* (18), *TFCP2L1* (17), *IPCEF1* (16), *MT1F* (14), *DPP6* (13), *CLDN1* (11), *PKHD1L1* (10), *SLC34A2* (9), *GABRB2* (8), *LRP4* (2), *LRRK2* (2), *TENM1* (1), *TFF3* (1). The correlation between *HGD*, *TPO*, *SLC26A4* and *DIO1* is shown in **Figure 3B**.

### RT-qPCR verification of risk gene set expression in clinical samples

**Figure 4:**
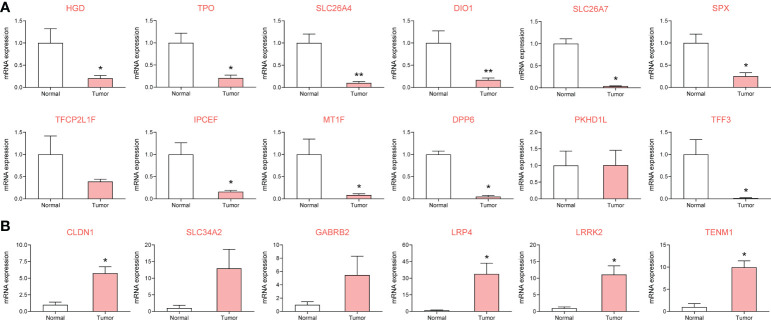
To investigate the expression of metastasis risk genes in papillary thyroid carcinoma (PTC) and adjacent clinical samples by RT-qPCR. 14 of which were significantly different. **(A)** DEGs downregulated in metastatic PTC compared with paracancellular tissues. **(B)** DEGs upregulated in metastatic PTC compared with paracancellular tissues. ^*^P< 0.05, ^**^P< 0.01 vs. normal group.

Based on eight patients with metastatic PTC, cancerous and normal adjacent tissues were collected. Expression of the risk-associated gene set was verified using RT-qPCR, and expression of the top four core risk-associated genes was verified using immunohistochemistry. In the pathological tissues from patients, 14 genes were differentially expressed in metastatic PTC, with statistically significant differences (*P* < 0.05). Primer sequences are shown in **Supplementary Table 1**, and RT-qPCR results are shown in **Figure 4**.

### Analysis of DEGs associated with relapse-free survival

**Figure 5:**
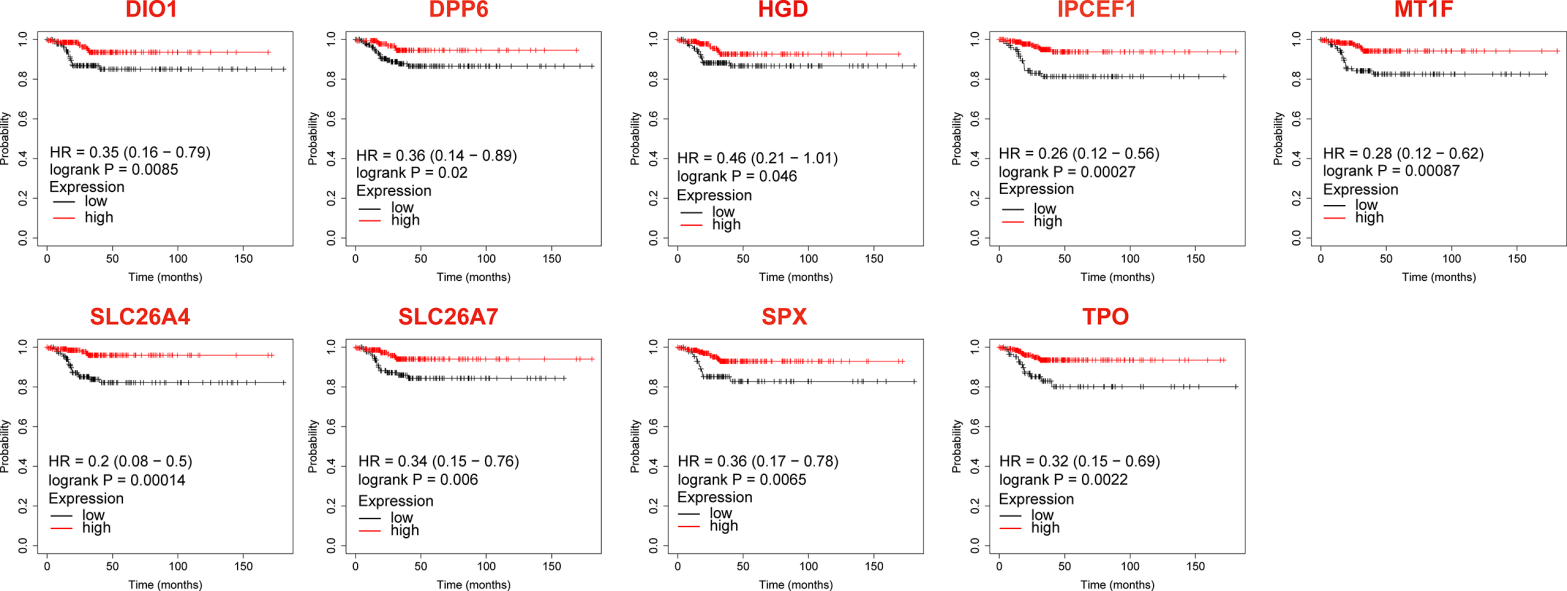
The Relapse-free survival of differentially expressed genes in thyroid cancer after validation by clinical samples.

Among the verified 14 genes with differential expression in the clinical samples, 9 DEGs significantly affected the RFS (*P* < 0.05, **Figure 5**) of patients with metastatic PTC, while the influence of 5 DEGs was not significant (*P* > 0.05).

### IHC verification of core risk gene set expression in clinical samples

**Figure 6:**
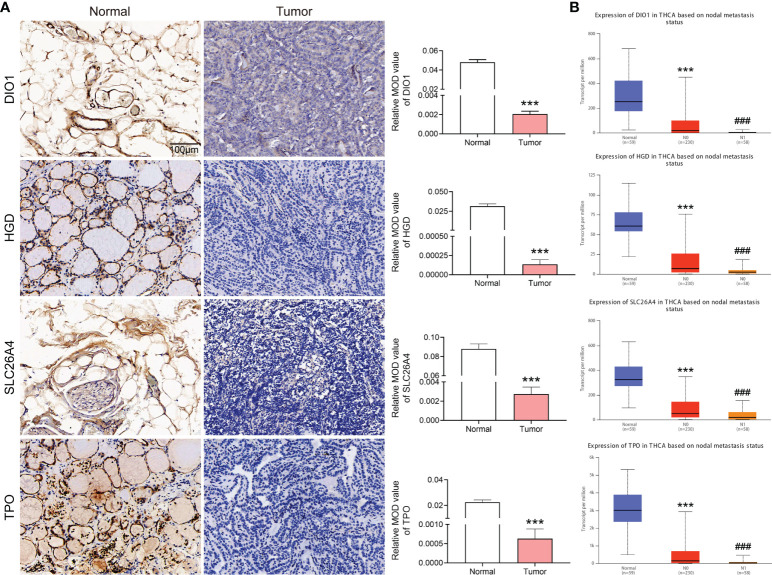
Expression of *DIO1*, *HGD*, *SLC26A4*, and *TPO* in metastatic papillary thyroid carcinoma (PTC) and adjacent normal tissues, and in different stages in the database. **(A)** Immunohistochemical staining of clinical samples, the expression of DIO1, HGD, SLC26A4 and TPO in normal and metastatic PTC. **(B)** *DIO1*, *HGD*, *SLC26A4*, and *TPO* expression in normal, PTC, and metastasized PTC based on TCGA database. N0: No regional Lymph node metastasis. N1: Metastases in 1–3 axillary Lymph nodes. ^***^P< 0.001 vs. normal group; *^###^P*< 0.001 *vs.* N0 group.

We analyzed *DIO1, HGD, SLC26A4* and *TPO* protein expression with IHC staining in metastatic PTC and its adjacent tissues. DIO1, HGD, SLC26A4 and TPO were significantly overexpressed in paracancerous tissues (**Figure 6A**). The judgment was based on the mean optical density (MOD) after Image-Pro Plus 6.0 analysis.

## Discussion

Although the 5-year survival rate of patients with PTC is more than 90%, approximately 30% of patients have recurrent PTC (18). The prognosis of patients with recurrent PTC is poor and often accompanied by LN metastasis. Without early intervention, the 5-year survival rate of patients with distal metastatic PTC is only 53.3% (19). Unfortunately, there currently are no reliable biomarkers for PTC risk (20), and the risk in patients cannot be assessed (21). However, a high recurrence rate and aggressive PTC are usually accompanied by severe thyroid epitaxy, LN metastasis, or distant metastasis (21). To identify PTC patients at a high risk of metastasis and recurrence at an early stage, this study focused on patients with metastatic PTC. The gene expression differences between metastatic PTC and normal tissues were analyzed using bioinformatics methods, and expression verification based on a large sample size was conducted using GEPIA, The Cancer Genome Atlas (TCGA), and other databases. Survival analysis was conducted to identify the genes associated with a risk of PTC metastasis. Clinical samples were collected for secondary validation of the screening results, to identify biomarkers that can be used to assess the risk of PTC metastasis.

In our study, 14 PTC metastasis risk-associated gene sets were screened, including four upregulated genes (*CLDN1*, *LRP4*, *LRRK2*, *TENM1*) and 10 downregulated genes (*DIO1*, *DPP6*, *HGD*, *IPCEF1*, *MT1F*, *SLC26A4*, *SLC26A7*, *SPX*, *TFF3*, *TPO)*. Among all the risk-related genes, *DIO1*, *HGD*, *SLC26A4* and *TPO* have the highest correlation with each DEG and significantly affect the RFS of patients with metastatic PTC. Comprehensive multi-data analysis showed that these four genes may be the key risk genes leading to PTC metastasis. Through further analysis of the core metastasis risk-associated genes, we found that the four DEGs (*DIO1*, *HGD*, *SLC26A4*, *TPO*) that highly correlate with each risk-associated gene are closely related to the transport and activation of iodine.

Further analysis of the relationship between iodine and PTC through literature search showed that abnormal iodine intake is a risk factor for PTC (22). However, existing studies have not fully clarified the relationship between iodine and PTC, though some studies have shown that PTC patients have higher iodine exposure (23–25) and that high iodine intake is a risk factor for PTC. Other studies (26, 27) have found that high iodine levels are not significantly correlated with the risk of PTC and only affect the growth and metastasis of PTC. Patients with PTC usually show high levels of urinary (25) and serum (28) iodine. Concomitantly, the level of thyroid-stimulating hormone (TSH) in PTC patients is higher than that in normal controls (29). Interestingly, iodine deficiency can lead to decreased thyroid hormone synthesis and secretion and thus increased TSH levels. This suggests that PTC patients have two seemingly contradictory pathological features occurring concomitantly, high iodine and high TSH levels, which might thus be predictors of poor prognosis for PTC (29–32).

To explore the causes of both high iodine and high TSH levels in patients with PTC, this study further analyzed the relationship between the identified core risk-associated genes for metastasis and PTC. A further analysis of the functions of downregulated core risk-associated genes in metastasized PTC revealed that DIO1 is a type 1 iodothyronine deiodinase that can convert T4 into T3, and T3 into T2. Notably, high free T4 is a risk factor for PTC (25). The possible mechanism through which low *DIO1* expression affects the occurrence and development of PTC is as follows: low *DIO1* expression leads to failed T4 de-iodization and a lack of free of T4 in the body. HGD participates in tyrosine metabolism. Tyrosine metabolism is related to thyroid hormone synthesis, and *HGD* expression in turn is highly correlated with *TPO*. The low expression of *HGD* might be involved in the occurrence and development of PTC by affecting *TPO* expression and thyroid hormone synthesis. *SLC26A4*, which encodes the pendrin protein located on the apical membrane of thyroid follicular cells, is a chloride/iodide ion transporter. SLC26A4 is a key protein that transports iodine into the follicle to synthesize thyroid hormones, and its low expression is an early event in thyroid tumorigenesis (33). *TPO* encodes thyroid peroxidase, which is synthesized by thyroid follicular cells and is present in the plasma membrane at the top of the follicular epithelial cells. Thyroid peroxidase can oxidize iodine in the follicular cells to activate it, providing activated iodine for thyroid hormone synthesis. *TPO* plays an important role in maintaining normal thyroid functions. With low expression of *TPO*, normal thyroid function cannot be maintained, and thyroid dysfunction is closely related to TC. In this study, we found that genes associated with iodine transport (*SLC26A4*), activated iodine (*TPO*), thyroid hormone synthesis (*HGD*), and disassembled free T4 (*DIO1*) are expressed at low levels in PTC, and their expression is further reduced in metastatic PTC (**Figure 6B**). The expression levels in normal, PTC, and metastatic PTC tissues are shown in **Supplementary Table 2**. In conclusion, iodine utilization in patients with metastatic PTC is blocked in many ways, including thyroid hormone synthesis. This indicates that patients with PTC are unable to utilize iodine in their body. Although serum iodine and urine iodine levels are elevated, thyroid hormone synthesis is still not possible, leading to elevated TSH levels. This study could explain the possible reasons for high serum iodine and TSH levels in PTC patients.

Previous studies have found that the level of iodine intake can affect thyroid function, but the relationship between iodine intake and TC is not clear (22); moreover, some studies (34) suggest that high iodine intake is an important risk factor for *BRAF* mutations and the subsequent development of PTC. Some studies (35) suggest that high iodine levels play a protective role in thyroid cells and attenuate acute *BRAF* oncogene-mediated miRNA dysregulation. Of concern, one study (36) found that the 10, 15 and 20-year survival rate of patients receiving iodine-131 is 56%, 45% and 40%. However, the 10 and 15-year survival rate of patients without iodine-131 treatment was found to be 10% and 6%. Iodine intake is closely related to the prognosis of TC, and the expression of *SLC26A4* and *TPO* in iodine-resistant PTC is lower (15). This further supports our speculation that serum iodine content and level of iodine intake might not fully reflect the effect of iodine on PTC and that the ability of the body to use iodine could be important for the occurrence and development of PTC. The found providing a new treatment idea for patients with metastatic PTC who do not respond to iodine radiotherapy. For example, iodine absorption and utilization should be first improved for iodine-refractory PTC to ensure the efficacy of radiotherapy.

**Figure 7:**
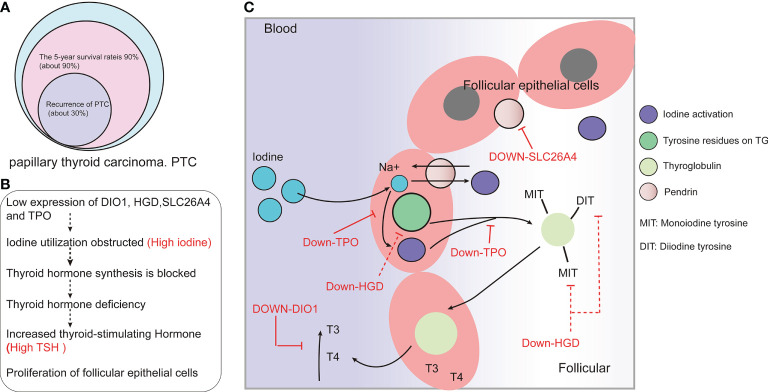
*DIO1*, *HGD*, *SLC26A4*, and *TPO* might be involved in the development of papillary thyroid carcinoma (PTC) by affects the absorption and utilization of iodine. **(A)** Prognosis of and recurrence rate in PTC patients. **(B)** Low expression of *DIO1, HGD, SLC26A4* and *TPO* may affect the synthesis and utilization of thyroid hormones. **(C)** The possible mechanism of iodine-resistant radiotherapy in patients with metastatic PTC: The low expression of *DIO1, HGD, SLC26A4*, and *TPO* leads to the inhibition of iodine recycling and thyroid hormone synthesis.

Although this study comprised a large-sample size analysis using GEO, GEPIA, TCGA, and other databases, in addition to utilizing pathological wax blocks from patients with metastatic PTC for RT-qPCR and immunohistochemistry verification, this was only a preliminary screen of a risk-associated gene set that might lead to PTC metastasis. As such, this set has not been quantitatively studied, and an evaluation of the risk of PTC metastasis based on the expression level of this gene set requires more clinical samples and quantitative studies based on each stage-based subgroup. A possible mechanism by which *DIO1*, *HGD*, *SLC26A4*, and *TPO* participate in the development of TC is shown in **Figure 7**.

## Conclusions

*CLDN1*, *LRP4*, *LRRK2*, *TENM1*, *DIO1*, *DPP6*, *HGD*, *IPCEF1*, *MT1F*, *SLC26A4*, *SLC26A7*, *SPX*, *TFF3*, and *TPO* affect the prognosis of patients with PTC and are risk-associated genes for PTC metastasis. Further, *DIO1*, *HGD*, *SLC26A4*, and *TPO* are potential core risk-associated genes. Our study will help assess the risk of metastasis in patients with PTC, so that patients at high risk of metastasis can be followed up early and closely.

## Data availability statement

The datasets presented in this study can be found in online repositories. The names of the repository/repositories and accession number(s) can be found in the article/**Supplementary Material**.

## Ethics statement

The studies involving human participants were reviewed and approved by Biomedical Research Ethics Committee of Zunyi Medical University Hospital. Written informed consent for participation was not required for this study in accordance with the national legislation and the institutional requirements.

## Author contributions

QZ designed the study and edited the manuscript. JL reviewed and revised the manuscript, HS proofread the manuscript. XB and TZ systemically revised the manuscript for important content. PL proposed the concept and designed the structure of the study. All authors contributed to the article and approved the submitted version.

## Funding

This work was supported by the Program for Excellent Young Talents of Zunyi Medical University (No.18zy-006) and the Basic Research Program of Guizhou Provincial Department of Science and Technology (Natural Science) [Grant No. qiankehejichu-ZK [2022]yiban599].

## Acknowledgments

We would like to thank Editage (https://www.editage.jp) for English language editing.

## Conflict of interest

The authors declare that the research was conducted in the absence of any commercial or financial relationships that could be construed as a potential conflict of interest.

## Publisher’s note

All claims expressed in this article are solely those of the authors and do not necessarily represent those of their affiliated organizations, or those of the publisher, the editors and the reviewers. Any product that may be evaluated in this article, or claim that may be made by its manufacturer, is not guaranteed or endorsed by the publisher.
