# Supplementary material for: Screening and validation of lymph node metastasis risk-factor genes in papillary thyroid carcinoma
Source: Front Endocrinol (Lausanne). 2022 Nov 7;13:991906. doi: 10.3389/fendo.2022.991906 (PMC9714616; doi:10.3389/fendo.2022.991906)
Supplement: Supplementary file 1 [file Table_1.docx]

Supplementary table 1. Eighteen papillary thyroid carcinoma transfer risk gene sets and internal reference primer sequences

| GENE | Forward primer | Reverse primer |  |  |
| --- | --- | --- | --- | --- |
| *ACTB* | CCTGGCACCCAGCACAAT | GGGCCGGACTCGTCATAC |  |  |
| *CLDN1* | CGAATTTGGTCAGGCTCT | GAAGGTGCAGGTTTTGGA |  |  |
| *DIO1* | CTGGCTGTCTGCTTGGA | GGTGCCTCCTCATTGCT |  |  |
| *DPP6* | GGGCCTGAACGACTACAA | GGATGAACAGACGCTACCA |  |  |
| *GABRB2* | AGTATCGGAAAGCTGGGTT | TCAGTCAAGTCAGGGATGG |  |  |
| *HGD* | GAGGATCGCCAAGTACCA | ACATTGAACGGGGAGACA |  |  |
| *IPCEF1* | ATGTGAAACCCATGCCTTA | GCGGAGAGGAGGAAATG |  |  |
| *LRP4* | CACATTCTCTTCCCTGCCT | GGTAAACAGCTCCGGTGA |  |  |
| *LRRK2* | GCCTCTGTTGATCGTCTTG | GCTTTGCATTGTACCTGGA |  |  |
| *MT1F* | TCTTCGCTTCTCTCTTGGA | TTTGCACTCTTTGCACTTG |  |  |
| *PKHD1L1* | CTGTTTGTGGGTCGCTCT | CACAGGGGTTCTGTTGCT |  |  |
| *SLC26A4* | ACAGGCTTTGATCTTGGG | AATTGGGGACCTGGGTAT |  |  |
| *SLC26A7* | CTTGGATTGAAATGAGTGAGG | GAAGAGTGTTTGATGGGAATG |  |  |
| *SLC34A2* | GAGTGGGGCAGATGGAG | ACAGGGAGGGCTTTTGTT |  |  |
| *SPX* | GCATTTTGTTTTCACTCAGC | CACCTTCCTTCCACATTCTT |  |  |
| *TENM1* | TTACCATAGAGGGGAGGGA | TGTTACCGATGAGCACCA |  |  |
| *TFCP2L1* | CATTGCCCCACTGTAAGC | GAAGGGAGAAAGGAGCCA |  |  |
| *TFF3* | AGGCACTGTTCATCTCAGC | AAAGTCTCAGGCACGAAGA |  |  |
| *TPO* | GCCATGTACGCCACGAT | GGGCAATCACTCCGCTT |  |  |

Supplementary table 2. Expression of DIO1, HGD, SLC26A4, and TPO in thyroid cancer obtained from the UALCN database.

| GENE | Comparison | Statistical significance |
| --- | --- | --- |
| *DIO1* | Normal-vs-N0 | 8.39E-07 |
|  | Normal-vs-N1 | 3.33E-16 |
|  | N0-vs-N1 | 9.62E-07 |
|  |  |  |
|  | Normal-vs-N0 | <1E-12 |
| *HGD* | Normal-vs-N1 | <1E-12 |
|  | N0-vs-N1 | 1.66E-08 |
|  |  |  |
|  | Normal-vs-N0 | 1.11E-16 |
| *SLC26A4* | Normal-vs-N1 | 1.62E-12 |
|  | N0-vs-N1 | 2.38E-06 |
|  |  |  |
|  | Normal-vs-N0 | 1.62E-12 |
| *TPO* | Normal-vs-N1 | <1E-12 |
|  | N0-vs-N1 | 4.00E-04 |

Note: N_0_: No regional lymph node metastasis; N_1_: Metastases in 1–3 axillary lymph nodes
